# Investigation on the Mechanical Properties of a Cement-Based Material Containing Carbon Nanotube under Drying and Freeze-Thaw Conditions

**DOI:** 10.3390/ma8125491

**Published:** 2015-12-14

**Authors:** Wei-Wen Li, Wei-Ming Ji, Yao-Cheng Wang, Yi Liu, Ruo-Xu Shen, Feng Xing

**Affiliations:** 1 Guangdong Key Provincial Durability Center for Marine Structure, Shenzhen Durability Center for Civil Engineering, Department of Civil Engineering, Shenzhen University, Shenzhen 518060, China; liweiwen@szu.edu.cn (W.-W.L.); weimingji163@gmail.com (W.-M.J.); 2141150214@email.szu.edu.cn (Y.L.); xingf@szu.edu.cn (F.X.); 2 Shenzhen Graduate School of Harbin Institute of Technology, Shenzhen 518055, China; ruoxushen@gmail.com

**Keywords:** carbon nanotube (CNT), cement-based material, mechanical property, durability, drying, freeze-thaw

## Abstract

This paper aimed to explore the mechanical properties of a cement-based material with carbon nanotube (CNT) under drying and freeze-thaw environments. Mercury Intrusion Porosimetry and Scanning Electron Microscopy were used to analyze the pore structure and microstructure of CNT/cement composite, respectively. The experimental results showed that multi-walled CNT (MWCNT) could improve to different degrees the mechanical properties (compressive and flexural strengths) and physical performances (shrinkage and water loss) of cement-based materials under drying and freeze-thaw conditions. This paper also demonstrated that MWCNT could interconnect hydration products to enhance the performance of anti-microcracks for cement-based materials, as well as the density of materials due to CNT’s filling action.

## 1. Introduction

Carbon nanotube (CNT) is a novel and nano-sized fiber with outstanding mechanical and physical properties. Theoretically, Young’s modulus, tensile strength and fracture strain of an individual CNT fiber can reach 1 TPa, 100 GPa and 15%, respectively [1,2]. Also, CNT has a high specific surface area with a value of up to 1000 m^2^·g^−1^ [3]. Due to these excellent mechanical and physical properties, CNT has been added to traditional materials, such as cement-based materials and polymers, for enhancement of their properties.

During the service life of concrete structures, durability is an important issue that must be considered. A number of severe environmental factors can deteriorate the mechanical performances of structural materials. For example, the hot dry environment is often considered to damage the durability of cement-based materials due to insufficient hydration, as well as the freeze-thaw cycling (FT) which can induce a series of internal micro-cracks in cement-based materials. In continental areas such as central Asia and parts of North America concrete could even experience both of these two extreme conditions (*i.e.*, cold weather in winter and hot climate in summer), and therefore designers need information about the performances of the materials when wet and frozen in winter as well as when hot and dry in summer [4]. Drying shrinkage and insufficient hydration are of great concern for concretes in hot and dry climates [4,5]. Besides, additional stress is generated in concretes by the FT effect and the materials become easy to crack and get surface scaling, which may speed up the ingress of detrimental ions and the degradation of mechanical properties [6,7,8,9,10]. In these areas, cement-based materials are required to have satisfactory performance in such severe environments.

Based on research, the mechanism for using CNT as a reinforcing component in cement-based material is based on its bonding, bridging and mesh filling effects [11,12,13], which redistribute the inner stress and inhibit the propagation of micro-cracks. The influences of CNT on the properties of cement-based materials from the aspect of durability can be divided into two series. On the one hand, some researchers have found that CNT is helpful for improvement of durability. Makar *et al.* [14] dispersed single-walled CNT (SWCNT) by sonication and observed from the cracks in SEM micrographs that SWCNT acted as bridges and reinforced the matrix; Han *et al.* [15] found that multi-walled CNT (MWCNT) could decrease the absorption coefficient, water permeability and gas permeability of cement-based materials. On the other hand, CNT was also reported to have detrimental effects on durability. Del *et al.* [16] detected a slight increase in porosity when CNT was added to cement-based material; the results also showed that the addition of CNT increased the degradation of concrete under carbonation and promoted the ingress of chloride ions; the reason for the higher corrosion level is that higher CNT dosage leads to higher conductivity and therefore a higher galvanic coupling effect from the CNT to the steel reinforcement. In summary, the reasons for getting the two contrary results from different works are complicated, but may include the different dispersion methods and dosages of CNT used in the studies.

During the use of CNT in cement-based materials, dispersion of CNT is an important step and influences the properties obtained. At the moment, dispersions of CNT into water by means of surfactant and sonication [17,18,19,20] are commonly used. Konsta-Gdoutos *et al.* [21] found that surfactant in combination with sonication could effectively disperse CNT in water. During the sonication, bubbles created by waves release high levels of energy and separate individual CNT from bundles. Besides, surfactant can be absorbed to the CNT surface and protects CNT from agglomeration. However, the surfactant applied was also found to act as an air entraining agent and resulted in adverse effects to the mechanical properties [22], while longer sonication pretreatment on the CNT may break its structure [23]. In 2013, Saravanan *et al.* [24] used a high energy ball milling process to disperse CNT into AA 4032 nanocrystalline matrix and found no structural damage in the CNT. Therefore, in this study, the ball milling dispersion method was used to disperse CNT during the casting of the samples.

Due to lack of a systematic study on the effect of CNT on the mechanical properties of cement-based materials under drying and freeze-thaw cycle conditions, this study is crucial to close the knowledge gap.

## 2. Experimental Program

### 2.1. Materials

**Figure 1:**
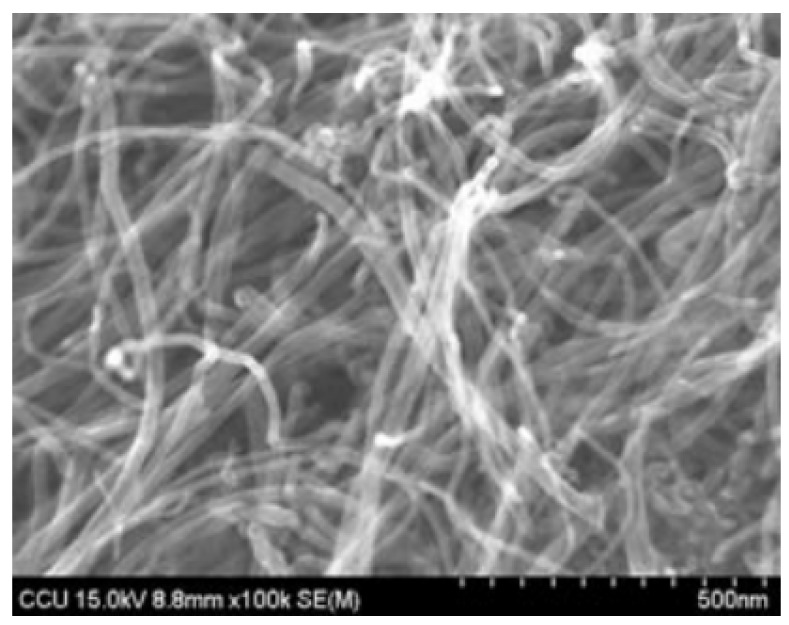
Scanning electon microscopy (SEM) image of multi-walled CNT (MWCNT).

The cement used in this study was Type I 42.5R Portland cement manufactured by Guangzhou Xinhe Co., Ltd. (Guangzhou, China). The fine aggregate used was standard sand provided by China ISO Standard Sand Co., Ltd. (Xiamen, China). The carbon nanotube was carboxylated MWCNT offered by Chengdu Institute of Organic Chemistry Research Institute (Chengdu, China). Its morphology and physical properties are shown in Figure 1 and Table 1, respectively. A polycarboxylate based superplasticizer was used to make sure that the cement composite specimens had similar workability.

### 2.2. Mix Proportions

Specimens with four different dosages of MWCNT, *viz.* 0%, 0.1%, 0.3%, 0.5% by weight of cement, were used in this study and they were denoted as “Control” and “Q-CNT”, respectively, where the “Q-CNT” indicates specimens with CNT. The content of superplasticizer used in each mix was determined on the basis of a slump diameter test, and the value was controlled at 160 (±5) mm in this study. Detailed mix proportions are listed in Table 2.

**Table 1:** Properties of the multi-walled CNT (MWCNT) used in this study.

Type	Diameter	Length	Purity	Specific Surface Area	-COOH
MWCNT	10–20 nm	10–30 μm	>95%	>120 m^2^·g^−1^	2 wt %

**Table 2:** Mix proportions (unit: g).

Specimen	Cement	Sand	Water	MWCNT	Superplasticizer
Control	100	100	45	0	0.2
Q1-CNT	100	100	45	0.1	0.3
Q3-CNT	100	100	45	0.3	0.4
Q5-CNT	100	100	45	0.5	0.6

### 2.3. Manufacture and Curing of Specimens

In the experiment, manufacturing and curing of samples followed the procedures listed below:

1. One g of MWCNT was mixed with 20 g of cement into an agate jar for the ball milling process, and the ball/mixture ratio (by mass) was 40:1;
2. The mixtures of cement and MWCNT were milled for 0.5 h with a QM-QX2 ball mill, manufactured by Nanjing Nanda Co., Ltd. (Nanjing, China), at a speed of 200 rpm for a homogeneous dry MWCNT-cement mixture;
3. Cement, sand, superplasticizer, and the pre-milled MWCNT-cement mixture were added into a NJ-160A cement mixer (manufactured by Wuxi Jianyi Experimental Instrument Co., Ltd., Wuxi, China), together with designated amount of water. The mixer was switched on at a quick speed for 3 min for fresh CNT/cement composite;
4. Fresh specimens were cast with steel molds into two different dimensions, which were 280 mm × 25 mm × 25 mm and 160 mm × 40 mm × 40 mm. The previous samples were cast for testing the drying shrinkage; the latter ones were cast to study the mechanical properties and analyses of the microstructure;
5. The specimens were demolded after 24 h from the cast and cured by two different regimes. The 280 mm × 25 mm × 25 mm samples designed for testing the drying properties were exposed to a drying condition with 20 °C and relative humidity (RH) of 50%; the 160 mm × 40 mm × 40 mm samples were exposed to a standard curing condition with 20 °C and RH of 95% until test.

### 2.4. Mechanical Properties Test

To study the effect of CNT on the mechanical properties of cement-based materials, the flexural strength and compressive strength of samples cured in a standard environment for 28 days were determined. The flexural and compressive tests were conducted according to ASTM C348 and ASTM C349, respectively.

### 2.5. Morphology and Pore Characteristics Study

Morphology and analyses of pore characteristics were carried out on samples cured in the standard condition for 28 days. The morphology of the composite was observed by SU-70 scanning electron microscopy (SEM) and the pore characteristics were analyzed by mercury intrusion porosimetry (MIP) (AutoPore IV 9500, manufactured by Micromeritics, Shanghai, China).

### 2.6. Properties under Drying Conditions

The drying shrinkage and water loss were explored on the 280 mm × 25 mm × 25 mm and 160 mm × 40 mm × 40 mm samples, respectively. The initial length (*L_0_*, in mm) and mass of specimen (*W_0_*, in g) were measured after demolding of the samples after the one day of curing in molds. Subsequently, samples were placed in the drying room with a controlled temperature of 20 °C and RH of 50% for drying curing. During the drying stage, the length (*L_n_*, in mm) and mass (*W_n_*, in g) of the specimens were tested every 24 h for 6 days. The lengths of the specimens were measured by a length gauge with an accuracy of 0.002 mm and the drying shrinkage *ε* was calculated following Equation (1). For the mass change, the water loss rate, *S*, was determined following Equation (2).

(1) $$\varepsilon =\frac{L_{0}-L_{n}}{L_{0}}\times 100\%$$

(2) $$S=\frac{W_{0}-W_{n}}{W_{0}}\times 100\%$$

The flexural strength and the compressive strength of specimens with dimension of 160 mm × 40 mm × 40 mm at 3, 7, and 28 days of the drying stage were also tested in this study, following the ASTM C348 and C349 standard.

### 2.7. Freeze-Thaw Cycling

Before exposing to FT regimes, the 160 mm × 40 mm × 40 mm specimens that were used for studying the anti-frost effect, experienced a 24-day standard curing and another 4-day immersion in water after demolding.

The FT regime was consistent with placement of specimens into a freezer for 16 h at a temperature of −20 °C and a subsequent immersion in water for 8 h at a temperature of 20 °C. The compressive strength of the specimens was tested after 30, 60, and 90 FT cycles, which was prescribed in GB/T50082 2009 as a key parameter for indicating the degree of degradation of cement-based materials under FT effect. The loss rate of compressive strength, Δ*f_c_*, was calculated based on Equation (3).

(3) $$\Delta f_{c}=\frac{f_{c0}-f_{cn}}{f_{c0}}\times 100\%$$

where *f_cn_* is the compressive strength (MPa) of specimen after *n* FT cycles; *f_c0_* is the compressive strength (MPa) of specimens constantly placed in the standard curing environment and tested together with the FT samples.

For all the mechanical tests, shrinkage and anti-frost tests carried out in this study, three replicates were measured for each mix and their average values were used to indicate the results. For the MIP measurement, two replicates were measured for each mix.

## 3. Experimental Analysis

### 3.1. Mechanical Strengths

**Figure 2:**
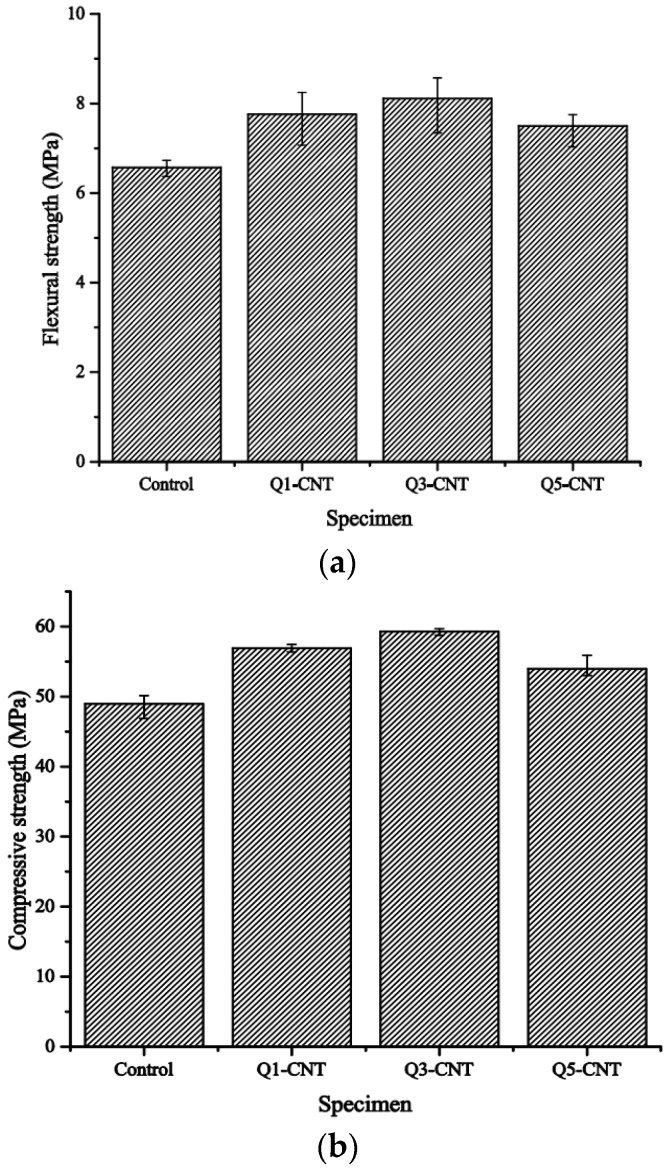
Flexural and compressive strengths of samples in standard curing. (**a**) Flexural strength; (**b**) Compressive strength.

Figure 2 shows the flexural and compressive strengths of specimens after 28-day standard curing.

As seen from the results, addition of the MWCNT led to an increase in both the flexural and compressive strengths. The largest improvements were observed in the Q3-CNT specimens, which were a 23.4% higher in the flexural strength and 21.1% higher in compressive strength. Further increase in the MWCNT content to a dosage of 0.5% led to a noticeable decrease in both the flexural strength and compressive strength in this study.

The improvements from CNT on the mechanical strengths of specimens have also been observed by many researchers and its mechanisms can be concluded as:

1. The effective network formed by MWCNT embedding firmly in the hydration products plays significant roles of bonding and bridging effects for the matrix [11]. This enhancement can provide higher stress when the crack goes straight toward the MWCNT reinforced zone area and prevent propagation of cracks at a high speed [25].
2. Carboxylic acid groups on the surface of MWCNT react with the calcium silicate hydrate and result in a strong embedding strength between the MWCNT and matrix [19].

By comparing the results obtained from this study with those published in the literature, as summarized in Table 3, it is noteworthy that in most cases CNT was dispersed by means of ultrasonication and surfactant. The extent of improvement in mechanical properties of cement composite varies from different studies, which can be attributed to the dispersion method, types and content of CNT used. In this study, CNT dispersed by the ball-milling dispersion method provided comparable enhancement in mechanical properties as good as with those dispersed by the ultrasonication and surfactant method. Therefore, results obtained from this study proved that the ball-milling method is an effective alternative for CNT dispersion in the cement matrix.

**Table 3:** Summary of different techniques used for CNT dispersion in the cement matrix and resulting improvement in mechanical properties.

Type and Content	Dispersion Technique	Improvement in Mechanical Properties	Researchers and Reference
0.05% MWCNT	Ultrasonication and superplasticizer	Compressive and flexural strength improved by 7% and 6%	Del *et al.* [16]
0.5% MWCNT	Functionalization with HNO_3_/H_2_SO_4_ mixture and direct mixing with cement	Compressive and flexural strength improved by 19% and 25%	Li *et al.* [19]
0.15% MWCNT	Direct mixing with cement	No improvement in compressive strength	Kim *et al.* [25]
0.1% SWCNT	Ultrasonication and surfactant	Compressive and flexural strength improved by 19% and 7%	Parveen *et al.* [26]
1% MWCNT	Direct mixing with cement	Compressive strength improved by 10%	Torkittikul *et al.* [27]
0.3% MWCNT	Ball-milling	Compressive and flexural strength improved by 23.4% and 21.1%	Li *et al.* (Present work)

### 3.2. Microstructure of Specimens

#### 3.2.1. Morphology of the CNT/Cement Composite

**Figure 3:**
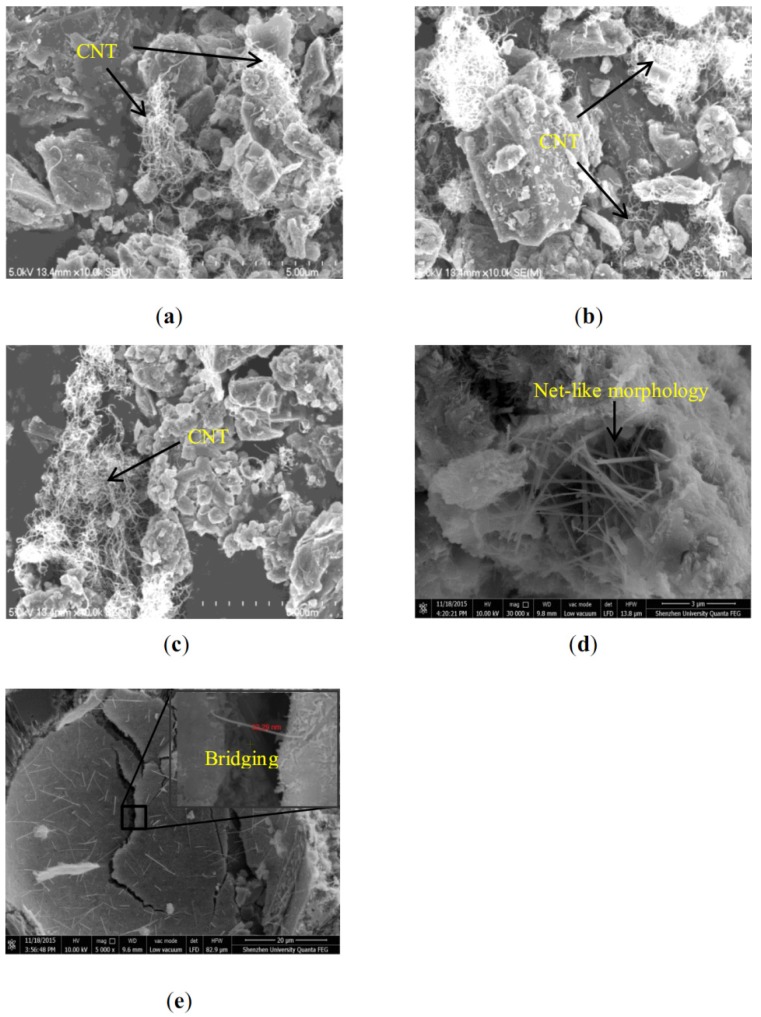
SEM images of the CNT/cement composite at an age of 28 days. (**a**) Q1-CNT ×10k; (**b**) Q3-CNT ×10k; (**c**) Q5-CNT ×10k; (**d**) Q3-CNT ×30k; (**e**) Q3-CNT ×5k.

SEM micrographs of the hydrated CNT/cement composite structure after 28-day curing are presented in Figure 3. As shown in Figure 3a–c, with the increase in content, MWCNT agglomerated much more obviously; this could be attributed to the Van der Waals' force between MWCNT. The agglomeration of MWCNT was the weak area of the matrix and could be the main reason for the reduction in mechanical properties (but still higher than that of control specimen), as shown in Figure 2. Figure 3d shows that MWCNT presented a net-like morphology in the pores of the cement hydration product, which was considered to be the most idealistic structure for MWCNT [25]. Generally, higher content of MWCNT requires more energy for dispersion during the milling process. Therefore, the ball milling method used in this study needs further improvement based on the dosage of MWCNT added. Figure 3e proves that parts of the MWCNT added in the Q3-CNT sample were well dispersed and the individual MWCNT was firmly wedged into the cement hydration products [28]. In the sub-area of Figure 3e with a magnification of 90 k, an individual CNT with a diameter of 32.29 nm was shown and interconnected hydration products. Besides, according to the activation theory [29], the ball milling process used, also charged the MWCNT surface and activated cement particles, which was helpful for interactive reaction between MWCNT and the cement hydration products.

#### 3.2.2. Pore Characteristics of the CNT/Cement Composite

With the initial study, it was found that the Q3-CNT specimen had the highest 28-day compressive strength. This mix was selected as the example for discussing the influence of MWCNT on pore characteristics and properties of the cement composite under drying regimes and FT cycles in the following discussions.

**Figure 4:**
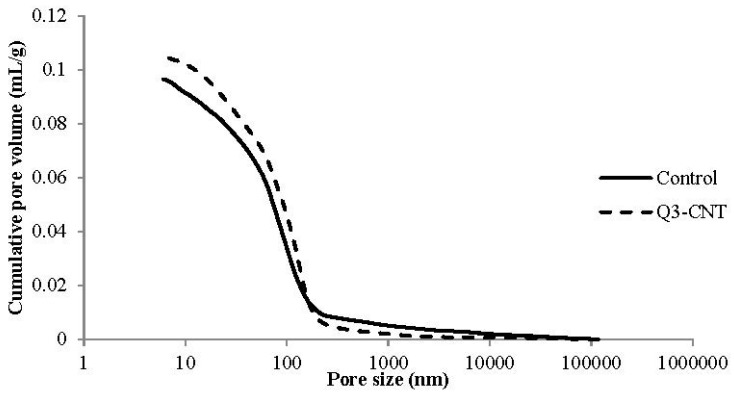
Pore size distribution curves of the Control and Q3-CNT specimens.

Table 4 and Figure 4 present pore characteristics for the Control and Q3-CNT specimens. As seen in Table 4, a slight increase in porosity was detected in the Q3-CNT at the 28th day compared to the control. The addition of MWCNT led to a decrease in pores with diameter smaller than 100 nm by 5.5%, pores with diameter bigger than 200 nm a decrease by 33.1%, and those in the range of 100–200 nm increased by 55.1%. It indicates that MWCNT addition can significantly reduce the volume of pores with a diameter of greater than 200 nm. Pores of this size are regarded as having a detrimental effect on the mechanical properties and durability of cement-based materials [30]. The increase in pore volume can be ascribed to filling of entangled MWCNT in larger voids, shown in Figure 3d, and dividing the pores into smaller sizes and forming a meshed cement mortar structure.

**Table 4:** Porosity and pore size distribution.

Specimen	Porosity (%)	Pore Size Distribution (mL/g)			
		<50 nm	50~100 nm	100~200 nm	>200 nm
Control	18.59	0.0316	0.0284	0.0274	0.0092
Q3-CNT	19.62	0.0317	0.0250	0.0425	0.0061

### 3.3. Drying Properties

#### 3.3.1. Water Loss of Specimen under Drying

**Figure 5:**
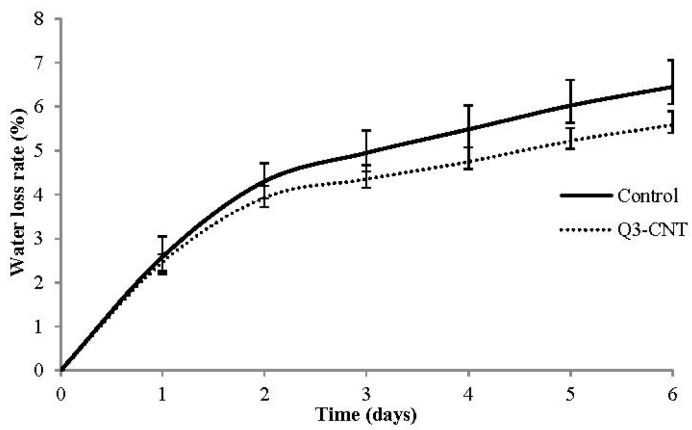
Average value of water loss rate.

Results of water loss for the control and Q3-CNT specimens are shown in Figure 5. The Q3-CNT specimen had a smaller water loss and a lower rate, compared with the control specimen. For both of the two samples, the water loss was comparatively at a high speed in the initial two days, and subsequently became stabilized. The addition of 0.3% MWCNT led to a reduction of lower water evaporation by 13.3% for the first six days of drying, indicating that there were less open pores in the Q3-CNT specimen.

#### 3.3.2. Drying Shrinkage

**Figure 6:**
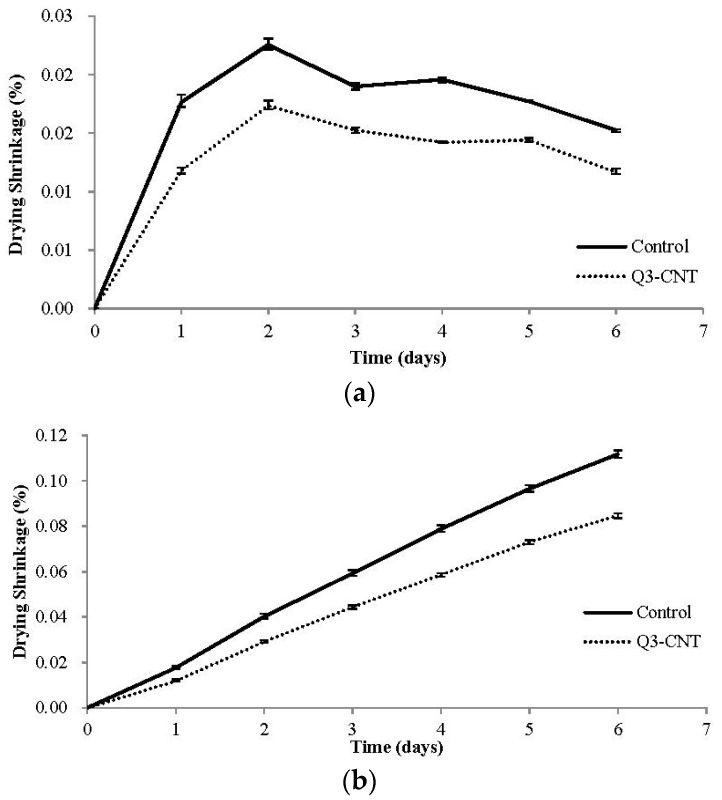
Development of drying shrinkage for the control and Q3-CNT specimens. (**a**) Average value of daily drying shrinkage; (**b**) Average value of cumulative drying shrinkage.

Figure 6 shows the development of daily and cumulative drying shrinkage of the control and Q3-CNT specimens in the studied drying stage. It can be seen that, the shrinkage of the Q3-CNT was at a much lower rate in comparison to the control specimen. Also, the drying shrinkage of both the two specimens was at a relatively high speed in the first two days, which gradually decreased in the succeeding four days. This development of drying shrinkage is consistent with the results of water lose rate as presented in Figure 5. At the end of the drying stage studied, the shrinkages of the Control and Q3-CNT specimens were 0.112% and 0.085%, respectively. Addition of 0.3% MWCNT led to an apparent reduction of 31.9% in the drying shrinkage.

#### 3.3.3. Development of Flexural Strength and Compressive Strength under the Drying Condition

**Figure 7:**
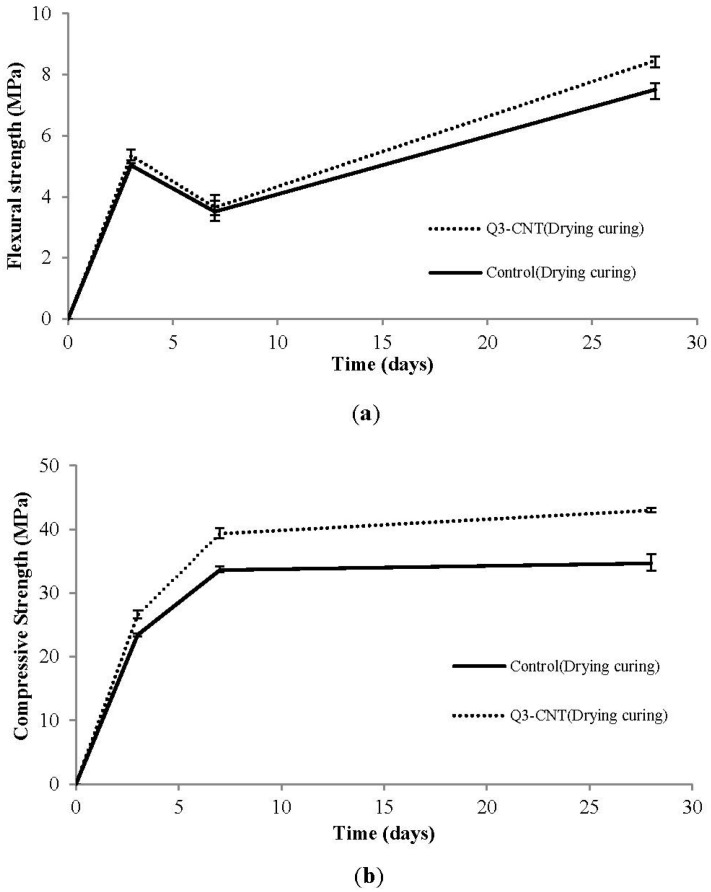
The flexural and compressive strengths of the control and Q3-CNT specimens under drying conditions (**a**) Average value of flexural strength; (**b**) Average value of compressive strength.

Figure 7 shows the flexural and compressive strengths of the control and Q3-CNT specimens placed under drying and standard conditions for 3, 7, and 28 days. During the drying, the flexural and compressive strengths of the Q3-CNT were higher than those of the control. The compressive strength of both the control and Q3-CNT obviously increased after the first 7 days under the drying condition, and a marginal improvement was detected at the 28th day. After the 28-day drying, the addition of MWCNT by a dosage of 0.3% led to an increase in flexural and compressive strengths by 24% and 12.4% respectively. In the development of flexural strength, there was a noticeable decrease from 3 days to 7 days, as seen in Figure 7a. Similar findings have also been reported by Al-Rub and Tyson *et al.* [17,31]. Based on the theory of Tyson *et al.* [31], the curing process would delay the formation of high stiffness C-S-H at the early days and hence delay the bonding mechanism between CNT and the cement matrix, which might explain the decrease in the flexural strength at the 7th day.

The mechanism of CNT/cement composites for the improvement in the drying process could be explained as follows:

1. The reasons for lower shrinkage could be attributed to less moisture migration and improved pore structures. For one reason, addition of MWCNT caused a reduction in the evaporation paths for water and the rate of water loss, preventing moisture migration to the outer surface and leading finally to lower drying shrinkage [32]. For another, MWCNT achieved the enhancement effect by increasing the amount of C-S-H gel of high hardness and improving pore structures [33].
2. The reasons for higher mechanical strength could be attributed to the improved pore structures and higher anti-cracking resistance. Both micro-cracks caused by a tension state on the surface [34] and insufficient hydration are detrimental to dried mortar and theoretically lead to reduction in compressive strength of the specimens. For one reason, during evaporation, water transports from the core area to the surface through pores of different dimensions, in which capillary pores act as a moving path and macro pores act as reservoirs for water evaporation. In the Q3-CNT specimen, there are less large pores on the surface zone and more water remains in the specimen [35], leading to further cement hydration. For another, the lower shrinkage has a significant effect on the mechanical behavior, as the development of micro-cracks due to volumetric changes is reduced. The bridging effect of MWCNT shown in SEM images will also lead to higher flexural and compressive strengths in the drying conditions.

### 3.4. Freeze-Thaw Cycling Test

**Figure 8:**
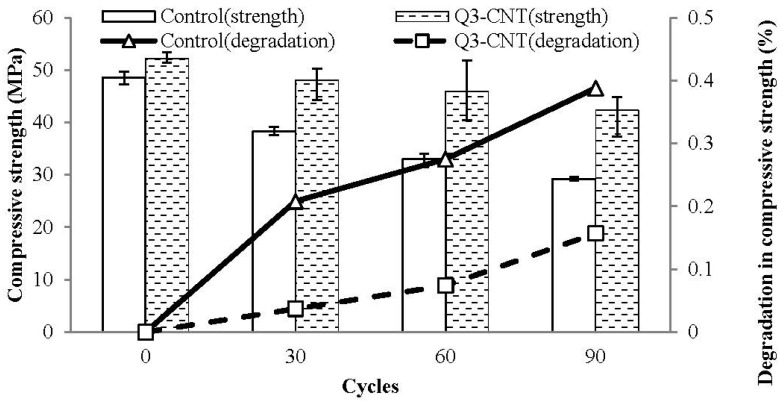
Degradation in compressive strength of the control and Q3-CNT specimens under freeze-thaw (FT) cycles.

In Figure 8, the histogram shows the compressive strength of specimens, and the line shows the degradation of the compressive strength under the effect of FT cycling. During the studied duration, the decrease in compressive strength for the Q3-CNT was lower than that of the control, which implies a better resistance of the CNT/cement composite against the FT effect.

(4) $$ln(\frac{T}{T_{0}})=-\frac{2\times \Delta G\times v_{w}}{\Delta h\times r}$$

Equation (4) is a derived Gibbs Thomson equation presented by Penttala [36]. In this equation, *T* (°K) is the temperature of pore liquid; *T*_0_ (°K) is the freezing temperature of the bulk liquid at a pressure of 1 bar; *ΔG* is the interfacial energy change between matrix and frozen liquid and between matrix and liquid during freezing; *v_W_* is the specific volume of water; *Δh* is the latent heat of fusion; and *r* is the pore radius. This equation can be further derived into the following Equation (5).

(5) $$T_{0}=T\times e^{\frac{2\times \Delta G\times v_{w}}{\Delta h\times r}}$$

From Equation (5), it can be concluded that the pore solution in bigger pores (a bigger “r” value) has a lower freezing temperature. Therefore, it can be deduced that during the decrease in temperature from modest room temperature to a frozen state, the solution in bigger pores freezes first. As a consequence, the unfrozen solution in smaller pores will be forced into the bigger pores via their connection channels, leading to a further increase in the diameter of ice crystals, which causes a further expansion [36]. This explains the better FT resistance of the MWCNT samples that have fewer bigger pores, as proved previously.

Besides, the better FT resistance of the MWCNT samples can also be attributed to the interlocking and bridging effects of the MWCNT in the hydrated cement paste.

Furthermore, the movement of gel water from smaller pores to bigger ones under the FT cycles will cause drying shrinkage of the gel structure [36]. This might be relative to the drying shrinkage tested during the drying curing of this study. The lower shrinkage has a significant effect on the FT resistance, as the development of micro-cracks due to volumetric changes is reduced. Therefore, fewer micro-cracks would prevent the penetration of the unfrozen solution and lead to smaller generation of osmotic pressure in the pore system.

## 4. Conclusions

This paper presents the results of investigation on the properties of cement mortar incorporated with MWCNT. Cement mortar samples with four different concentrations of MWCNT (0 wt %, 0.1 wt %, 0.3 wt %, and 0.5 wt %) were designed for this study. The morphology of the MWCNT in the cement matrix and its effect on the flexural strength, compressive strength, porosity, drying shrinkage, and FT resistance properties were investigated. The following conclusions were drawn from the study.

- Carbon nanotube dispersed by the ball-milling method in cement matrix provided enhanced mechanical properties. At a dosage of 0.3% of cement, the carbon nanotube/cement composite has the highest mechanical strength in this study.
- With addition of carbon nanotube to the cement mortar, pores with a diameter of bigger than 200 nm are likely to be refined to 50–100 nm; the disconnection of pores was also improved.
- Samples incorporated with carbon nanotube have a better performances than the pure cement mortar under drying and the freeze-thaw conditions. This is attributed to pores refinement, drying shrinkage reinforcement, and the bridging effect of the carbon nanotube.
